# Parkin (PARK 2) Mutations Are Rare in Czech Patients with Early-Onset Parkinson's Disease

**DOI:** 10.1371/journal.pone.0107585

**Published:** 2014-09-19

**Authors:** Ondrej Fiala, Daniela Zahorakova, Lenka Pospisilova, Jana Kucerova, Milada Matejckova, Pavel Martasek, Jan Roth, Evzen Ruzicka

**Affiliations:** 1 Department of Neurology and Centre of Clinical Neuroscience, 1st Faculty of Medicine and General University Hospital, Charles University, Prague, Czech Republic; 2 Institute of Neuropsychiatric Care (INEP), Prague, Czech Republic; 3 Department of Pediatrics and Adolescent Medicine, 1st Faculty of Medicine, and General University Hospital, Charles University, Prague, Czech Republic; 4 Department of Pathology and Molecular Medicine, Thomayer's University Hospital, Prague, Czech Republic; Hospital General Dr. Manuel Gea González, Mexico

## Abstract

**Objective:**

The aim of the study is to determine the frequency of parkin allelic variants in Czech early-onset Parkinson's disease patients and healthy controls.

**Methods:**

A total of 70 early-onset Parkinson's disease patients (age at onset ≤40 years) and 75 controls were screened for the sequence variants and exon rearrangements in the parkin gene.

**Results:**

Parkin mutations were identified in five patients (7.1%): the p.R334C point mutation was present in one patient, four patients had exon deletions. The detected mutations were observed in the heterozygous state except one homozygous deletion of the exon 4. No mutations were obtained in control subjects. A novel sequence variant p.V380I (c.1138G>A) was identified in one control. Non-pathogenic polymorphisms p.S167N and p.D394N were seen in similar percentage in patients and controls, polymorphism p.V380L was almost twice as frequent in controls as in patients.

**Conclusions:**

Our study contributes to the growing body of evidence on the low frequency of the parkin mutations in the early-onset Parkinson's disease suggesting the potential role of other genes in the pathogenesis of the disease.

## Introduction

Parkinson's disease (PD) is a chronic progressive neurodegenerative disorder, clinically characterized by resting tremor, rigidity, and bradykinesia, as well as non-motor impairment such as cognitive deficit or autonomic dysfunction [1]. The prevalence of PD is about 0.3% in the entire population and more than 1% in the population over the age of 60 years [2]. The mean age at onset (AAO) of PD is usually between 60–70 years [3], however 3–5% of all patients with PD have onset before the age 40 years [4]. This rare form of the disease is referred as early-onset PD (EOPD); the incidence of EOPD is estimated 0.5 per 100 000 per year [5]. The clinical phenotype of EOPD differs from classic PD in several features such as slower disease progression, more frequent occurrence of dystonia, marked sleep benefit, excellent treatment response, and early development of levodopa-induced dyskinesia and motor fluctuations [4], [6]. Among several genes whose mutations are associated with autosomal recessive EOPD, parkin mutations were shown as the most common [7].

Parkin is one of the largest human genes spanning approximately 1.38 Mb. It consists of 12 coding exons separated by large intronic regions. The parkin gene encodes a 465-amino acid protein, which contains a ubiquitin-like domain at the N terminus and a RING (Really Interesting New Gene) domain composed of three RING finger motifs (RING0, 1, and 2). RING1 and 2 are separated by a sequence without any recognizable domain structure named IBR (in between-RING) [8]. Parkin exhibits E3 ubiquitin ligase activity and mediates the ubiquitination of a number of proteins, thus targeting them for proteasomal degradation. Parkin has been reported to influence mitochondrial fusion, mitophagy and mitochondrial transport through ubiquitylation of mitofusin, voltage dependent anion channel 1 (VDAC1) and Miro/Milton complex, respectively. Parkin has also been shown to be important in the regulation of mitochondrial biogenesis [9].

More than 180 pathologic allelic variants of the parkin gene have been described with half of them being located in the region spanning exons 2 to 4. The exon rearrangements account for more than 50% of all parkin mutations [10], [11]. Lücking and colleagues reported the parkin mutations in 50% of familial as well as 18% of sporadic EOPD cases [7]. In contrast, other studies have shown a pathogenic mutation frequency as low as 1.6–8.6% [12]–[14]. The prevalence of the parkin mutations vary widely across studies, possibly due to a population-specific variability in allele frequencies. The aim of this study is to determine the frequency of parkin allelic variants in Czech EOPD patients and healthy controls.

## Materials and Methods

### Patients and controls

A total of 70 unrelated Czech patients (47 males, 23 females; mean age 47.7 years) with EOPD (AAO ≤40 years) were recruited from the Movement Disorders Center, Prague, Czech Republic. The diagnosis of PD was based on the UK Brain Bank diagnostic criteria, not including the presence of family history as an exclusion criterion [15]. Controls were 75 healthy unrelated individuals (52 males, 23 females; mean age 45.4 years) recruited from the Department of Blood Transfusion, General University Hospital, Prague, Czech Republic. Written informed consent was obtained from all individuals. Phenotypic data were assessed by personal interview and neurological examination, both in patients and in controls. All controls had normal neurological examination and negative family history for parkinsonism. Family history was defined as positive if the proband had at least one affected first- or second-degree relative. Personal interview and clinical evaluations of EOPD patients included the severity of PD (Hoehn and Yahr stage; H-Y), the presence of dystonia, hallucinations, dysautonomia (hyperhidrosis, hypersalivation, urinary dysfunction, constipation), and sleep benefit, as well as the response to dopaminergic therapy and the occurrence of levodopa-induced dyskinesia and motor fluctuations. The study was approved by the ethics committee of the General University Hospital, Prague, Czech Republic.

### Molecular analysis

For genetic analysis, a venous blood sample was collected and genomic DNA was isolated from peripheral blood leukocytes using a standard salting-out procedure. All 12 exons of the parkin gene were amplified from the patient's genomic DNA by the polymerase chain reaction (PCR). For amplification, previously described primers were used [16]. All fragments were analyzed in both directions on a 3500xL Genetic Analyzer (Applied Biosystems). Parkin allelic variants were numbered relative to Genbank mRNA sequence (accession number NM_004562).

Controls were screened for the sequence variants identified in patients using high resolution melt analysis (HRM). The primers used for HRM were the same as those used to generate the PCR products for sequencing in patients. Reference samples of known genotypes were included into each sample group. The samples were then analyzed in Light Scanner instrument (Biofire Defence) using a melt range of 65°C to 98°C. Acquired data were analyzed by the supplied Call-IT 1.5 software using the auto group and high sensitivity settings.

To identify exon rearrangements in the parkin gene, we analyzed gene dosage in patients and controls using multiplex ligation-dependent probe amplification (MLPA) using SALSA MLPA kit P051-C3-0313 and P052-C2-0313 (MRC-Holland). The MLPA assay was performed according to the manufacturer's protocol. The fragments were analyzed on a 3130 Genetic Analyzer (Applied Biosystems) with the fragment analysis software Gene Mapper 4.0. For each sample, the relative peak area was calculated and compared with controls using the Coffalyser v.131211 software.

The identified allelic variants were classified according to terminology of the Human Gene Mutation Database (HGMD). The prediction of the pathogenicity was performed using electronic tools MutPred and SIFT. The genotype frequencies in the general population were calculated from data of the Exome Variant Server (European American population).

### Statistical analysis

The Fisher Exact Test was used to assess differences in frequency of parkin allelic variants between patients and controls. Odds ratios (OR) are given with their 95% confidence intervals (CI). Statistical analysis was performed using the GraphPad Prism 6 software.

## Results

### Clinical characteristics of patients

Clinical characteristics of EOPD patients are summarized in Table 1. The mean AAO was 35.0±4.9 years, the average disease duration amounted to 12.3±8.0. The mean H-N stage was 2.1±0.9. Dystonia occurred in more than half of the patients (55.7%), the half of the individuals (50.0%) referred marked sleep benefit. A relatively high percentage of patients reported hallucinations (18.6%). The most common sign of dysautonomia was hyperhidrosis (57.1%), followed by hypersalivation (42.9%), urinary dysfunction (28.6%), and constipation (27.1%). An excellent response to dopaminergic therapy was reported by two thirds of the cases (68.6%), levodopa-induced dyskinesia and motor fluctuations were registered in more than half of the patients (51.4% and 70.0%, respectively). Dyskinesia had developed after a mean interval of 5.4 years ±4.1 after commencing dopaminergic treatment, motor fluctuations appeared even earlier (4.7 years ±4.1). A positive family history was present in 10 (14.3%) individuals.

**Table 1:** Clinical characteristics of patients (n = 70).

Characteristics	Patients
Male/Female *(% male)*	47/23 (67.1%)
Age at onset *(mean* ± *SD) years*	35.0±4.9
Age at examination *(mean* ± *SD) years*	47.4±8.4
Disease duration *(mean* ± *SD) years*	12.3±8.0
H-Y stage *(mean* ± *SD)*	2.1±0.9
Positive family history	10 (14.3%)
Dystonia	39 (55.7%)
Hallucinations	13 (18.6%)
Hyperhidrosis	40 (57.1%)
Hypersalivation	30 (42.9%)
Urinary dysfunction	20 (28.6%)
Constipation	19 (27.1%)
Sleep benefit	35 (50.0%)
Excellent response to dopaminergic therapy	48 (68.6%)
Levodopa-induced dyskinesia	36 (51.4%)
Latency of dyskinesia *(mean* ± *SD) years*	5.4±4.1
Motor fluctuations	49 (70.0%)
Latency of motor fluctuations *(mean* ± *SD) years*	4.7±4.1

### Genotypic characteristics and frequency of allelic variants of the parkin gene

Genotypic characteristics of the patients and controls are shown in Table 2, the frequency of allelic variants of the parkin gene is reported in Table 3. Previously described polymorphisms p.S167N and p.D394N were seen in similar percentage in patients (7.1%, 10.0%) and controls (9.3%, 8.0%). Polymorphism p.V380L was almost twice as frequent in controls (25.3%) as in patients (14.3%). A novel sequence variant p.V380I (c.1138G>A) was identified in one control (1.3%). One patient (1.4%) and three controls (4.0%) were carriers of two different polymorphisms (p.S167N + p.D394N, p.S167N + p.V380L, p.V380I + p.D394N). Disease-causing mutations (HGMD classification) p.A82E, p.R334C, and p.R402C occurred in three patients (4.3%) and one control (1.3%); from those subjects, a shared mutation with another polymorphism (p.A82E + p.D394N, p.V380L + p.R402C) was detected in one patient (1.4%) and one control (1.3%). Exon deletions were identified in four patients (5.7%). All allelic variants were observed in the heterozygous state, except two homozygous polymorphisms (p.V380L in patient, p.S167N in control) and one homozygous deletion (Ex4del). The patient with this mutation was also heterozygous for deletion of exons 2–3 and heterozygous for the p.V380L polymorphism; his family history was positive (Table 2, Patient 3). No statistically significant difference was found in the frequency of parkin allelic variants between patients and controls (Table 3).

**Table 2:** Genotypic characteristics of patients and controls.

Individual	Sequence variant/mutation	Positive family history
Patient 1	p.A82E het + p.D394N het	no
Control 1, Patient 2	p.S167N het + p.D394N het	no
Patient 3	p.V380L het + ex2-3del het + ex4del hom	yes
Control 2	p.S167N hom + p.V380L het	no
Control 3	p.V380I het + p.D394N het	no
Control 4	p.V380L het + p.R402C het	no
Patient 4–7, Control 5–10	p.S167N het	patient 4
Patient 8	p.R334C het	no
Patient 9–16, Control 11–26	p.V380L het	patient 9,10
Patient 17	p.V380L hom	no
Patient 18–22, Control 27–30	p.D394N het	no
Patient 23	p.R402C het	no
Patient 24	ex2del het	no
Patient 25	ex1-2del het	no
Patient 26	ex2-5del het	no

het - heterozygous, hom- homozygous.

**Table 3:** Frequency of allelic variants of the parkin gene in patients (n = 70) and controls (n = 75).

Allelic variant	Zygosity	HGMD class	SIFT/MutPred	EVS genotype frequency	Frequency in patients	Frequency in controls	Fisher *P*-value	OR	95% CI
**SNPs/point mutations**									
p.A82E	het	DM	Tolerated/Low risk	0.51%	1 (1.4%)	0 (0.0%)	0.489	3.26	0.13–81.40
p.S167N	het	DP	Tolerated/Very low risk	3.74%	5 (7.1%)	6 (8.0%)	1.0	0.89	0.26–3.04
p.S167N	hom	DP	Tolerated/Very low risk	0.05%	0 (0.0%)	1 (1.3%)	1.0	0.35	0.01–8.80
p.R334C	het	DM	Tolerated/Medium risk	NA	1 (1.4%)	0 (0.0%)	0.483	3.26	0.13–81.40
p.V380L	het	DP	Tolerated/Very low risk	28.51%	9 (12.9%)	19 (25.3%)	0.062	0.44	0.18–1.04
p.V380L	hom	DP	Tolerated/Very low risk	2.84%	1 (1.4%)	0 (0.0%)	0.483	3.26	0.13–81.40
p.V380I	het	NA	Tolerated/Low risk	NA	0 (0.0%)	1 (1.3%)	1.0	0.35	0.01–8.80
p.D394N	het	DP	Tolerated/Very low risk	8.53%	7 (10.0%)	6 (8.0)%	0.775	1.28	0.40–4.01
p.R402C	het	DM	Damaging/High risk	0.51%	1 (1.4%)	1 (1.3%)	1.0	1.07	0.07–17.49
**Exon rearrangements**									
Ex1del	het	DM	NA	NA	1 (1.4%)	0 (0.0%)	0.483	3.26	0.13–81.40
Ex2del	het	DM	NA	NA	4 (5.7%)	0 (0.0%)	0.052	10.22	0.54–193.50
Ex3del	het	DM	NA	NA	2 (2.9%)	0 (0.0%)	0.231	5.51	0.26–116.90
Ex4del	het	DM	NA	NA	1 (1.4%)	0 (0.0%)	0.483	3.26	0.13–81.40
Ex4del	hom	DM	NA	NA	1 (1.4%)	0 (0.0%)	0.483	3.26	0.13–81.40
Ex5del	het	DM	NA	NA	1 (1.4%)	0 (0.0%)	0.483	3.26	0.13–81.40

CI - confidence interval, DM - disease causing mutation, DP - disease-associated polymorphism, EVS - Exome variant server, het - heterozygous, HGMD - Human gene mutation database, hom - homozygous, NA - not available, OR - odds ratio.

## Discussion

The basic clinical characteristics of Czech EOPD patients do not differ from previous descriptions of EOPD phenotype [4], [6], [17]. Our results confirmed a high occurrence of dystonia (55.7%), marked sleep benefit (50.0%), an excellent response to dopaminergic therapy (68.6%) as well as high rate of the levodopa-induced dyskinesia (51.4%) and motor fluctuations (70.0%) with early development after commencing the dopaminergic treatment (dyskinesia  = 5.4 years; motor fluctuations  = 4.7 years). The signs of dysautonomia (hyperhidrosis, hypersalivation, urinary dysfunction, constipation) were also common, hallucinations occurred in a rather high percentage of patients (18.6%). The following non-motor symptoms also prevail in late-onset PD [18] having negative impact on health-related quality of life [19].

Polymorphisms p.S167N and p.D394N were as common in patients as in controls. Although both polymorphisms are classified according to HGMD as disease-associated, meta-analytic studies did not show the association between these polymorphisms and PD risk [20], [21]. Polymorphism p.V380L was seen almost twice more frequently in controls than in patients. It corresponds with the recent meta-analysis demonstrating association of the p.V380L polymorphism with decreased risk for PD [22], despite the fact that the p.V380L variant is listed as disease-associated polymorphism in HGMD.

A novel allelic variant p.V380I (c.1138G>A) was identified in one control. Prediction analysis using MutPred and SIFT estimated a low probability of pathogenic effect (Table 3).

One patient was a carrier of the p.A82E sequence variant (labeled as disease causing mutation in HGMD). This variant is very likely to be non-pathogenic as its presence has been previously observed in healthy controls [23]; it has a benign prediction analysis, and no effect of the p.A82E variant on the cellular distribution of the protein in vitro [24]. Even though the p.R402C allelic variant has been predicted as damaging and classified as disease causing mutation in HGMD (Table 3), its pathogenic status is equivocal since it has been also detected in healthy subject [25]. In our sample, heterozygous p.R402C variant was present in one patient and one control. The minor-allele frequency in European American population is estimated at 0.26% according to the Exome Variant Server (EVS). Therefore, we assume that p.R402C is a non-pathogenic silent substitution.

Mutations of the parkin gene were identified in five patients (7.1%): the p.R334C point mutation was present in one patient (1.4%), four patients (5.7%) had exon deletions. All deletions contained exon 2 encoding ubiquitin-like domain [26], which is crucial for a normal function of the protein and prevents its autoubiquitination [27]. No exon deletion was obtained in controls suggesting the possible pathogenic role of exon rearrangements in the etiology of EOPD. The detected mutations were observed only in the heterozygous state except one homozygous deletion of the exon 4. With regard to recessive inheritance of parkin type of EOPD, the presence of the homozygous or two compound heterozygous parkin mutations is required to cause the disease. Although the clinical significance of single heterozygous parkin mutations remains unclear, partial evidence indicates that heterozygous mutations also contribute to PD risk [28]. Unaffected carriers of heterozygous parkin mutations show presynaptic dopaminergic dysfunction in the striatum [29], [30] and hyperechogenicity of the substantia nigra [31], [32]. These preclinical changes suggest that heterozygous parkin mutations might contribute to the pathogenesis of EOPD.

The incidence of PD increases with age; it is five times higher at the age between 40–49 years than in the range of 30–39 years [5]. EOPD is considered a disease with AAO ≤40 years [8]. Nevertheless, many studies applied higher AAO, typically ≤45 [33]–[35] or ≤50 years [36]–[39]. The application of various criteria for AAO may cause misinterpretation when comparing data (e.g. the percentage of identified mutations) among studies. Thus, we adopted the most strict AAO criteria (≤40 years) in order to preserve a more phenotypically homogeneous patient's sample at the expense of its size.

The relatively small size of our patient's sample (n = 70) was a substantial limitation for the statistical analysis. Although there was no statistically significant difference in the frequency of parkin allelic variants between patients and controls, noticeable contrast can be seen in two cases: a twice higher frequency of heterozygous p.V380L polymorphism in controls (p = 0.062; OR = 0.44; 95% CI = 0.18−1.04) and an exclusive occurrence of heterozygous deletion of exon 2 in four patients (p = 0.052; OR = 10.22; 95% CI = 0.54−193.50).

In our previous pilot study [40], using less strict criteria for the AAO (≤45 years), we reported parkin mutations and polymorphisms among 45 Czech patients with EOPD, however their clinical significance was unclear due to the lack of control group. In the present case-control study, we are able to address this issue. Since we found previously a high percentage of the parkin polymorphisms in patients (34.9%), we suggested these polymorphisms as possible risk factors for EOPD. In the light of the new results presented here, it appears however that the detected parkin polymorphisms (p.S167N, p.V380L, p.D394N) are not risk factors for EOPD.

Overall, our study contributes to the growing body of evidence on the low frequency of the parkin mutations in the EOPD [12], [13], [41], [42], although it does not corroborate with the other studies indicating high prevalence of these mutations [7], [35]. Furthermore, we have identified a low percentage of EOPD familiar cases positive for mutations in the parkin gene (only one case; 10%) in comparison to previous observations [7]. Possible explanations for this discrepancy could be a population-specific variability in allele frequencies, different AAO criteria, various ratios of sporadic and familiar cases, and different criteria for determining the allelic variants as mutations. Interestingly, the study in Polish EOPD patients similarly showed low frequency of the parkin mutations (3.8%) [14]. It may suggest the potential role of other genes in the pathogenesis of EOPD in Slavic population.
